# Blood glucose sensing by back gated transistor strips sensitized by CuO hollow spheres and rGO

**DOI:** 10.1038/s41598-022-26287-8

**Published:** 2022-12-19

**Authors:** Milad Farahmandpour, Hassan Haghshenas, Zoheir Kordrostami

**Affiliations:** 1 Department of Electrical and Electronic Engineering, Shiraz University of Technology, Shiraz, Iran; 2 Research Center for Design and Fabrication of Advanced Electronic Devices, Shiraz University of Technology, Shiraz, Iran

**Keywords:** Biophysics, Biotechnology, Engineering, Electrical and electronic engineering

## Abstract

In this work, a highly sensitive flexible glucose sensor based on a field effect transistor (FET) has been fabricated. It is shown that the proposed flexible transistor can be used as new non-enzymatic blood glucose test strips. CuO hollow-spheres decorated with reduced graphene oxide have been synthesized using the hydrothermal method. The shells of the hollow micro-spheres are formed by nanostructures. The synthesized nanostructured hollow micro-spheres (rGO/CuO–NHS) are deposited on a flexible PET substrate between interdigitated electrodes as the channel of a back gate transistor. The channel concentration and the FET bias are optimized so that the sensor exhibits extremely low limit of detection and high sensitivity. The combination of selective porous CuO hollow spheres and the high surface to volume ratio of their nanostructured shells with the high mobility and high conductivity rGO led to faster and higher charge-transfer capability and superior electro-catalyst activity for glucose oxidation. The glucose-dependent electrical responses of the sensor is measured in both resistive and transistor action modes. The amplification of the current by the induced electric field of the gate in the proposed FET-based biosensor provides advantages such as higher sensitivity and lower limit of detection compared to the resistive sensor. The flexible glucose sensor has a sensitivity of 600 μA μM^−1^ and a limit of detection of 1 nM with high reproducibility, good stability, and highly selectivity. The high accuracy response of the biosensor towards the real blood serum samples showed that it can be used as a test strip for glucose detection in real blood samples.

## Introduction

**Figure 1:**
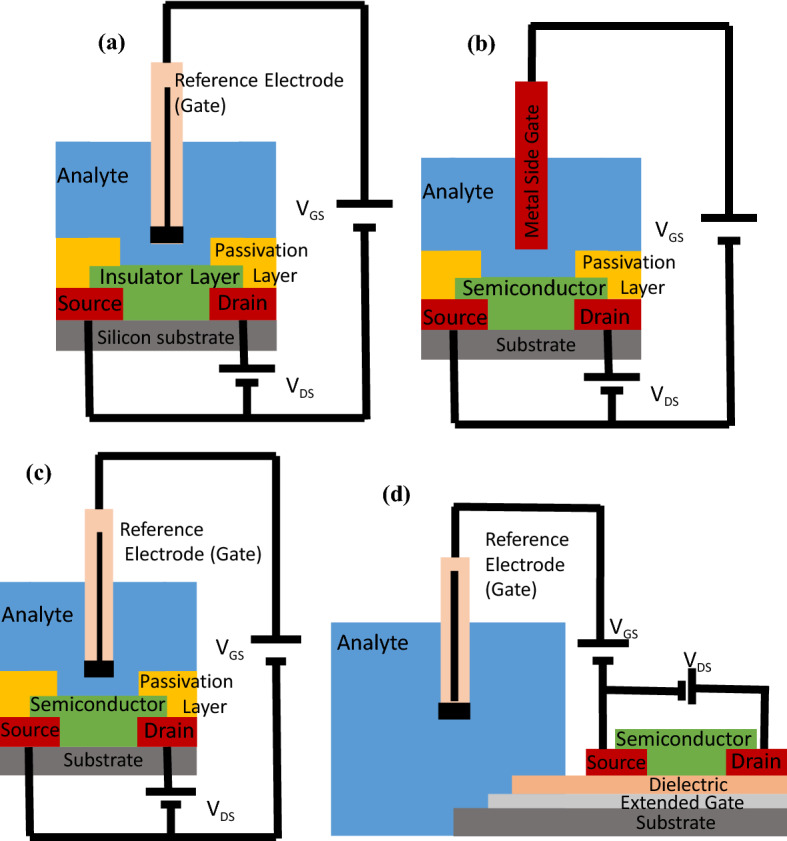
Schematic of the electrochemical glucose detection methods of the FET type.

Electronic devices have always shown their potential to be used as biosensors. According to report of the International Diabetes Federation, over 693 million people worldwide will be suffering from diabetes by 2045^1^. Diabetes arises from insufficiency or ineffectiveness of insulin secretion by the pancreas that puts the global population health in risk and can lead to serious complications to body organs^2^. Millions of people around the world need to test their blood sugar daily. Such a high demand for blood glucose testing has developed glucose biosensors as the most important tool, accounting for about 85% of the total biosensors market^2^. This indicates the requirement of developing wireless, low-cost, portable, and highly sensitive and selective biosensors using new methods and new nano-materials. Extensive research has been done on the development of advanced technologies for glucose sensing in recent years, such as optical, electrochemical, and microwave sensing. These technologies have been increasingly utilized to monitor the diabetic patients but they suffer disadvantages and problems such as low sensitivity, instability, and complex manufacturing processes. Electronics can help solving almost all of the mentioned problems. This work overcome these challenges by presenting the concept of the field effect transistor (FET)-based glucose sensor with a highly sensitive channel which is more sensitive than the available glucometers and it can be widely used in portable, wearable, or implantable^3,4^ applications for continuous and real-time measurement of the glucose. Electrochemical sensors are the most commonly used glucose sensors because of their good performance, simplicity, and low cost. They use three electrodes that are working, counter, and reference electrodes. To be used in practical applications, the reference electrode needs to shrink which degrades the sensitivity and stability of the sensor. Other disadvantages of this method is the large amount of solution in the electrochemical cell in the laboratories, effects of temperature, toxicity and pH of the cell on the biological protein molecules, problems in accurate detection of low glucose concentrations, unstable glucose detection process and the need for a potentiostat instrument^5,6^. We propose the fabrication of the FET-type glucose sensors, which exhibit higher sensitivity and selectivity and lower limit of detection compared to the other conventional methods. There are four types of FET based biosensors as shown in Fig. 1. Based on Fig. 1a, the ion-sensitive FET (ISFET) can be formed as a modified MOSFET where the metal gate is replaced by a system containing an insulation layer, an electrolyte solution, and a reference electrode. This method suffers from high noise levels and poor stability^7,8^. Figure 1b indicates the side-gate field-effect transistor (SGFET). The SGFET can be achieved by an on-substrate metal side-gate^9^. However, the integration of the Ag/AgCl electrode on a chip is complicated. Also, the process of the generation of the electric field by the gate is challenging and complex. This method still suffers from the disadvantages of the electrochemical sensors. This sensor does not fully benefits from the FET performance since it does not use the gate oxide. According to Fig. 1c, the electrolyte-gate FET (EGFET), is composed of an Ag/AgCl electrode to produce a stable gate voltages through the solution and adjust the operating point of the FET to get the optimum response^8,10^. The extended-gate FET (ExGFET) is a MOSFET with an extended gate electrodes shown in Fig. 1d. The extended gate is immersed in the solution while the other parts of the structure stay dry. This method has the advantages of easy fabrication process and comfortable exchange of the sensing electrode because MOSFET is located in dry environment. However, it suffers from the problems of the electrochemical method.

In recent years, glucose sensors have used various channel materials such as carbon nanotubes, metal oxides, and graphene. In general, metal oxides such as NiWO_4_, NiO, ZnO, CuO, MnO_2_, MoS_2_, Fe_2_O_3_, SnO_2_, TiO_2_, Ag_2_O, and In_2_O_3_ have been used by researchers for glucose sensing^11–24^. Copper oxide (CuO) is widely used as a very favorable material for glucose sensing. That is because of its low fabrication cost, easy synthesis, good availability in nature, and high electrocatalytic properties for glucose oxidation^23–25^. The CuO is a semiconductor with a band-gap of 1.2 eV. CuO nanostructures have been widely studied in glucose sensing with higher sensitivity, faster response, more stable detection and better selectivity over other metal oxides^22,23,26^.

Many glucose sensors have been fabricated by using functional materials, including carbon-based nanostructures (carbon nanotubes and graphene)^25,27–29^. Among them, graphene has been successfully used in sensors for its interesting physical properties, high thermal conductivity, high surface-to-volume ratio, excellent mobility, high transparency, flexibility, high electrochemical activity, and chemical stability^30–42^. Recently researchers showed that the composition of various forms of graphene such as reduced graphene oxide, nanoplate graphene, and nitrogen-doped graphene with different nanostructures such as Cu nano-flower, copper oxide, zinc oxide, gold and silver nanoparticles improves the sensitivity of the developed material for glucose sensing applications^43–49^.

Many materials used in the literature take advantage of the coating or decoration with other metal oxides, metals, and polymers to increase the sensitivity of the sensor. Ahmad et al. have reported ZnO nanorods decorated with CuO nanoparticles used in a non-enzymatic electrochemical glucose sensor. The CuO/ZnO hybrid material exhibited high sensitivity due to the higher surface-to-volume ratio. Also CuO possess a key role in excellent catalytic properties which enhance glucose electrochemical oxidation^23^. CuO nanostructures have been used in non-transistor-based sensors for enzyme-free glucose detection. Our research group has previously proposed a highly sensitive non-enzymatic glucose sensor based on hollow nanoporous CuO/ZnO microstructures on a glassy carbon electrode. The synthesized material exhibited high performance due to the enhancement of electrochemical reactivity and improvement in the glucose electrochemical oxidation^50^. Ahmad et al. reported a non-enzymatic flexible FET-based glucose sensor using NiO quantum dots and ZnO nanorods on Polyimide substrate which improved the sensing performance toward different glucose concentrations^51^. Also, Ahmad et al.^52^ and Mishra et al.^53^ have reported CuO nanowires (NWs) and nano-leaves used in an enzyme-free glucose sensor by growing CuO structures on the sensing electrodes. In this paper, we have proposed a new back-gated FET-based glucose sensor capable of producing an amplified readout signal that can detect molecules such as glucose in biological environments. Most of the glucose sensors use the glucose oxidase (GO) enzyme that boosts the detection selectivity and sensitivity. However, the enzymes are not suitable and stable for long-term glucose monitoring systems^54^. We propose a back-gate FET with CuO hollow-spheres decorated with reduced graphene oxide (rGO/CuO-NHS) as the channel to improve the selectivity, sensitivity, and stability of the glucose sensors. The novelty of the present work lies in the use of CuO hollow spheres with nanostructured shells improved by rGO (in terms of limit of detection and sensitivity) as the channel of a back gate FET on a flexible substrate. The results confirm the high-performance of the proposed bioelectronic glucose sensor that exhibiting promising sensing parameters without using enzymes.

One of the demands of the future technology market is developing of the smart sensor systems integrated into the Internet of Things (IoT)^55–57^. The proposed FET biosensor can easily convert the current changes to voltage changes so that it can be digitized and transmitted. The data can be received by a smart phone or can be accessed through internet.

## Results and discussion

### Sensor design and sensing mechanism

**Figure 2:**
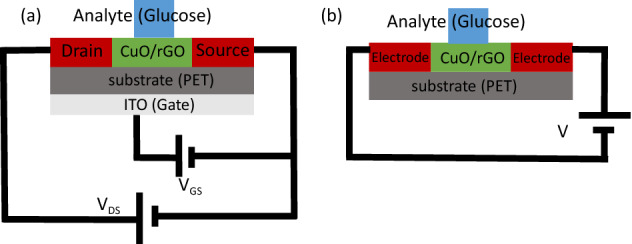
Schematic of the proposed flexible back-gate glucose sensor, (**a**) transistor mode. (**b**) Resistive mode.

To overcome the mentioned shortcomings, we propose back-gate bio-electronic FET (BGFET) shown in Fig. 2a as a very high-performance alternative for other types of electrochemical sensors. When the gate electrode is floated, the device reduces to a resistive sensor as shown in Fig. 2b. However, by applying the gate voltage, the sensor performance improves.

There are some features and capabilities in the FET biosennsor that are missing in electrochemical sensors such as:

1. The transistor action that provides the amplification of the signal. The gate voltage can control the channel and amplifies the current. This increases the sensitivity and lowers the limit of detection.
2. The strong electric field that fastens the transport of the generated electrons. This reduces the response time.
3. The FET itself is an electronic device that can be miniaturized and integrated into portable and wearable devices.
4. The measurement does not need potentiostat but instead a biasing circuit of the FET and the current measurement circuit can perform the measurements which have much lower cost than the potentiostat.

**Figure 3:**
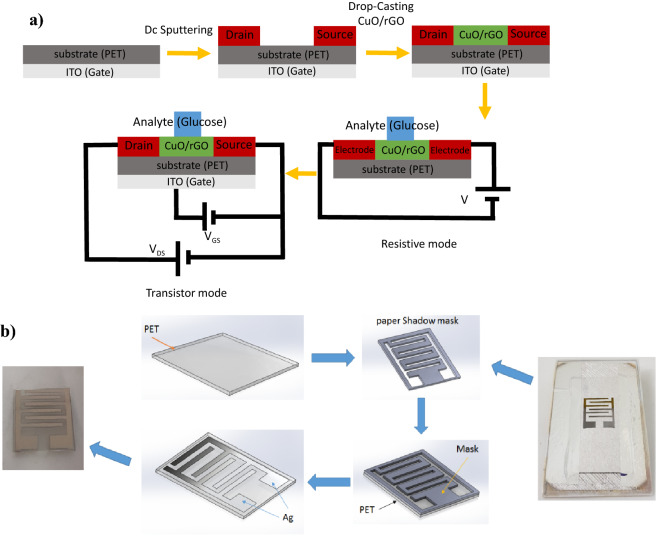
(**a**) Schematic of the fabrication process of the rGO/CuO-NHS BGFET sensor for glucose detection in both resistive and transistor modes. (**b**) Steps of fabricating the silver interdigitated electrodes on the PET substrate with a shadow mask.

The schematic of the fabrication process of the rGO/CuO-NHS BGFET sensor has been shown in Fig. 3a.

The BGFETs enjoy the advantages such as a lower amount of solution needed for testing, small dimensions, well-established fabrication process, portability, accurate and stable glucose detection, high input resistance, low noise, low power consumption, wide dynamic ranges, high sensitivity, high selectivity, compatibility with digital technologies such as wireless and smart-sensing technology and easy integration with other bio-electronic devices.

### Structural and morphological properties of material

An advanced glucose sensor needs to maximize the electrocatalytic oxidation of glucose. Recently it is found that combination of two materials can achieve this goal. A highly sensitive material (CuO in this case) with high electrochemical activity and another material that enhances the electrocatalytic activity of glucose oxidation (rGO in this case). CuO has a highly specific surface area, good electrochemical activity and the capacity of promoting electron transfer reactions at a lower overpotential which are severely required for the development of non-enzymatic glucose sensors. Since it is previously recognized that the electrochemical property of the active materials is directly related to their morphologies^58^.

**Figure 4:**
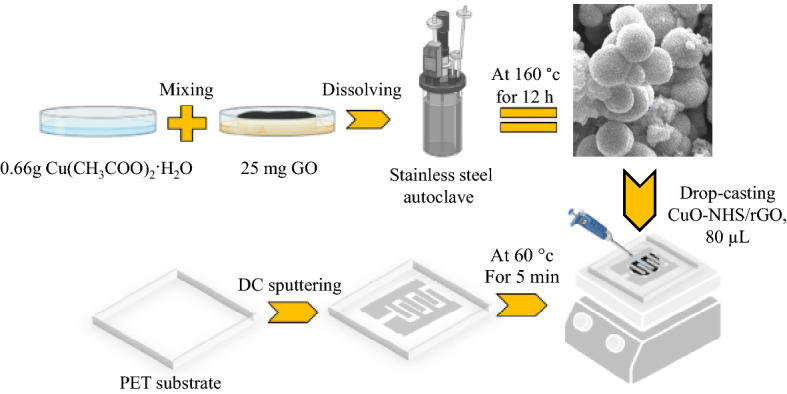
Schematic illustration of the material synthesis process and the fabrication of the rGO/CuO–NHS BGFET sensor.

The proposed morphology of the CuO has also a considerable effect on the improved the sensitivity of the sensor as well. Redox activity of the sensitive materials is another key factor leading to improved electrochemical properties during glucose detection. Composite material with multiple redox pairs can enhance electrocatalysis and promote redox reactions^59^. We have used graphene oxide which is reduced during the process of formation of CuO hollowspheres. Our experience shows that this kind of combining CuO and rGo provides very high electrocatalytic activity of glucose oxidation. The specific combination that we have used displays an obvious promotion for electrocatalytic oxidation of glucose that directly affects the sensor performance parameters such as sensitivity and detection limits. This combination has also proved its high selectivity toward glucose. The schematic illustration of the rGO/CuO-NHS synthesis process and its deposition on the fabricated BGFET sensor is shown in Fig. 4.

**Figure 5:**
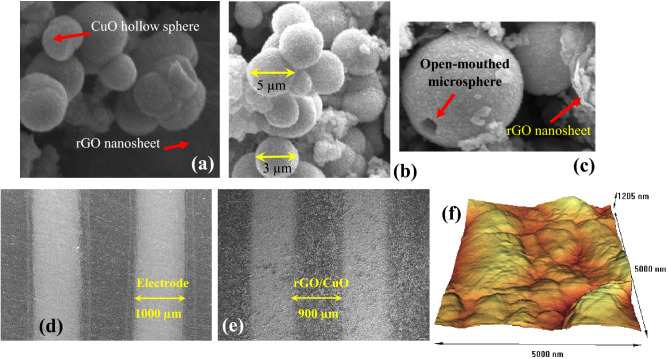
The SEM images of surface morphology of rGO/CuO–NHS. (**a**) The as-prepared rGO/CuO–NHS, (**b**) a large number of uniform CuO microspheres and their sizes, (**c**) an open-mouthed microsphere decorated with rGO nanosheets, (**d,e**) The SEM images of electrodes before and after deposition of rGO/CuO-NHS, (**f**) the AFM pattern after deposition of rGO/CuO–NHS between the electrodes.

The surface morphology of the rGO/CuO-NHS was investigated by Scanning Electron Microscopy (SEM) and the atomic force microscopy (AFM) are shown in Fig. 5. Figure 5a indicates the CuO microspheres decorated with reduced graphene oxide.

Figure 5b shows a large number of uniform microspheres. The average diameter of the synthesized CuO hollow spheres is nearly 4 µM, and the rough shells of the spheres are composed of nanoscale particles. Figure 5c shows an open-mouthed microsphere of rGO/CuO–NHS formed by Ostwald ripening process. Based on the image, it is found that the CuO hollow microspheres are scattered on the surface of layered and crumpled rGO nanosheets.

CuO hollow microspheres have been formed by Ostwald ripening process during the hydrothermal synteses^60^. In this process, the hollow CuO spheres consist of irregular tiny particles aggregated on the surface of the spheres’ shell.

According to the above-discussed results, the formation mechanism could be explained for the CuO/rGO sample as follows. Since Cu(CH_3_COO)_2_·H_2_O is disbanded in the DI water, CH_3_COO^−1^ and Cu^2+^ started to separate. CH_3_COO^−^ reacted with DI water to produce OH^−1^ and CH_3_COOH at 160 °C. The Cu^2+^ and OH^−1^ make Cu(OH)_2_ which after the H_2_O is removed from the CuO^60–62^.

The SEM images of electrodes before and after deposition of rGO/CuO–NHS between the electrodes are shown in Fig. 5d,e respectively. The SEM image presented in Fig. 5 indicates the spherical morphology of the prepared CuO microstructures and the presence of the rGO sheets is confirmed. The synthesized CuO microspheres have the mean size of 2–5 µm. It can also be seen that small nanoscale structures are self-aggregated and oriented themselves to form the larger spheres. The AFM micrograph of rGO/CuO on electrodes is depicted in Fig. 5f with the peak height of the 1205 nm. The surface analysis showed a surface structure composed of some mountains like structures with can be attributed to the hollospheres. Since the rGo sheets are combined with CuO hollowspheres during the synthesis process, pure dome-like structures are not observed on the surface.

**Figure 6:**
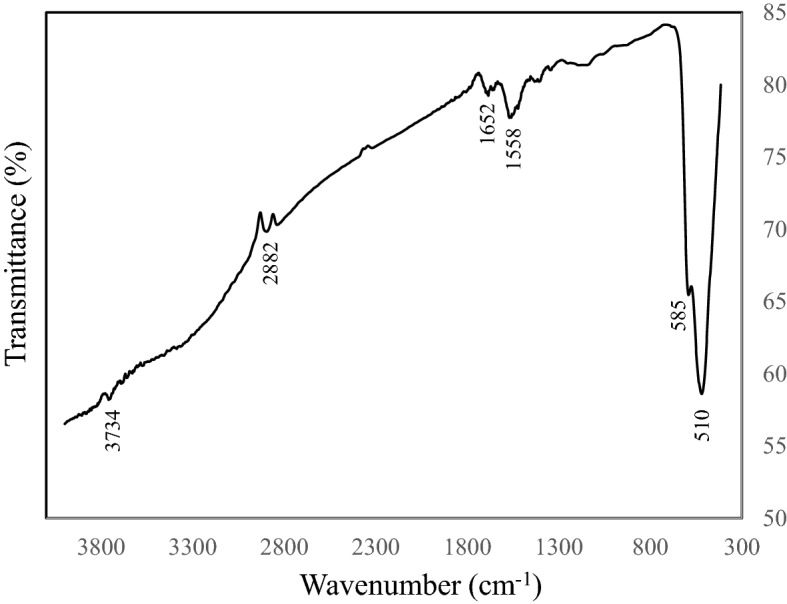
FTIR spectrum of the synthesized rGO–CuO–NHS.

Figure 6 shows Fourier transform infrared (FTIR) spectroscopy. FTIR spectroscopy was employed to characterize and identify various chemical bons and functional groups. The absorption peaks at 510 cm^−1^ and 585 cm^−1^ are assigned to the Cu–O bonds and confirm the formation of CuO. The characteristic peaks at 1558 cm^−1^ and 1652 cm^−1^ show the presence of C=C and C–O (carbonyl) stretching vibration, respectively. This confirms the incorporation of rGO into the product. The peak obtained at 2882 cm^−1^ can be attributed to the C–H bond and the the corresponding OH peak could be seen at about 3734 cm^−1^. As the GO reduces to rGO during the synthesis process, the oxygen-containing groups are extracted. That’s why the peak intensities related to the stretching vibrations of C=O, C–O–C, C–O and OH have been significantly reduced or disappeared in the spectrum.

**Figure 7:**
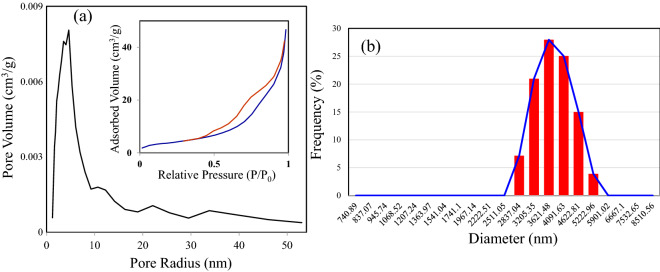
(**a**) The pore size distribution and the corresponding Nitrogen adsorption–desorption isotherm curves (inset) obtained from BJH and BET methods. (**b**) The size distribution of rGO/CuO–NHS obtained from DLS measurements.

The surface area, the pore size, and the pore size distribution of the rGO/CuO–NHS were obtained from the nitrogen adsorption–desorption isotherms using the Brunauer–Emmett–Teller (BET) and Barrett–Joyner–Halenda (BJH) methods as shown in Fig. 7a. As presented in the inset of in Fig. 7a, the isotherms correspond to type III, with H3 type hysteresis loop. The surface area and the pore volume of rGO/CuO–NHS are respectively 14.58 m^2^/g and 3.35 cm^3^/g with a mean pore diameter of 19.79 nm. The incorporation rGO has increased the surface area of the sensitive material. This is due to the formation of exfoliated rGO sheets and their homogeneous distribution in the material. The corresponding pore size distribution of the synthesized sample as presented in Fig. 7b evidenced the presence of a broad size distribution in the size range of 1.2–53 nm indicating that the synthesized samples are mesoporous in nature.

The size distribution of rGO/CuO–NHS was explored using dynamic light scattering (DLS) measurements. The number of particles and their size distribution are shown in Fig. 7b. According to particle size distribution measurements, the average particles size is around 3621 nm which is in correspondence with the SEM images of the synthesized material.

**Figure 8:**
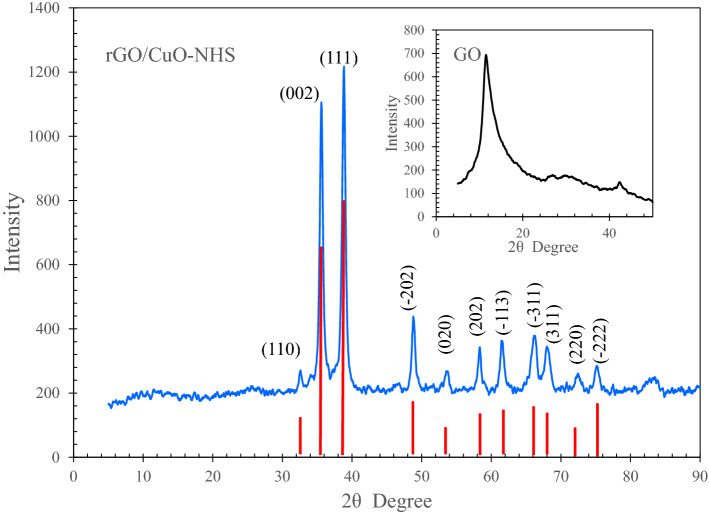
XRD pattern of the synthesized rGO/CuO-NHS. The inset is the XRD of GO.

The X-ray diffraction (XRD) spectroscopy of rGO/CuO-NHS is represented in Fig. 8. As for the CuO, the diffraction peaks are consistent with the peaks of the copper oxide standard PDF#48-1548. The peaks at 2θ values of 32.6°, 35.6°, 38.8°, 48.7°, 53.5°, 58.3°, 61.6°, 66.3°, 67.9°, 72.4°, and 75.3° correspond to (110), (002), (111), (− 202), (020), (202), (− 113), (− 311), (220), (311), and (− 222) planes of CuO. The XRD pattern verifies the formation of the CuO in the synthesized material. Although the diffraction peaks of the crystal planes of CuO can be clearly found, the (001) diffraction peak of the graphene oxide has been disappeared in the XRD pattern of the composite material which demonstrates that the GO was reduced successfully to rGO during the synthesis process. However, the typical peak of rGO located at 25° was not observed, which is due to the little amount of rGO and its incorporation into the product. The same result has been obtained in Ref.^60^. This happened due to the presence of less agglomerated, disordered stacking of the rGO sheets inside the composite matrix^63^.

**Figure 9:**
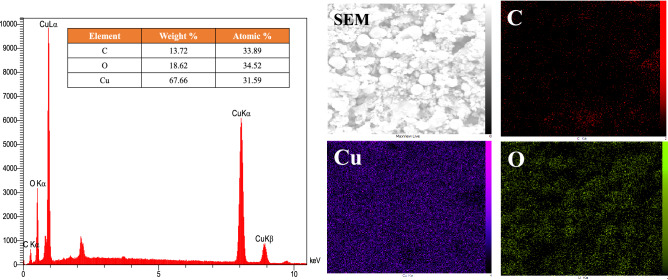
EDX and elemental mapping of rGO/CuO–NHS.

The Energy-dispersive X-ray spectroscopy (EDX) shown in Fig. 9, verifies the presence of the desired elements. The EDX spectrum of the rGO/CuO exhibits the presence of strong signals of Cu, and O (the SEM of the scanned region has also been shown). The Cu content is about 67.66%, O is 18.62% and C is 13.72%. The mapping results of the zone are also presented in which the distributions of Cu and O elements are the very same. The obtained results for C agree with the the little amount of rGO incorporated into the product. It is deduced that the elements O, C, and Cu are uniformly distributed which indicates that the CuO and rGO are evenly dispersed in the whole rGO/CuO-NHS.

### Electrical characteristics of the fabricated flexible BGFET glucose sensor:resistive mode

**Figure 10:**
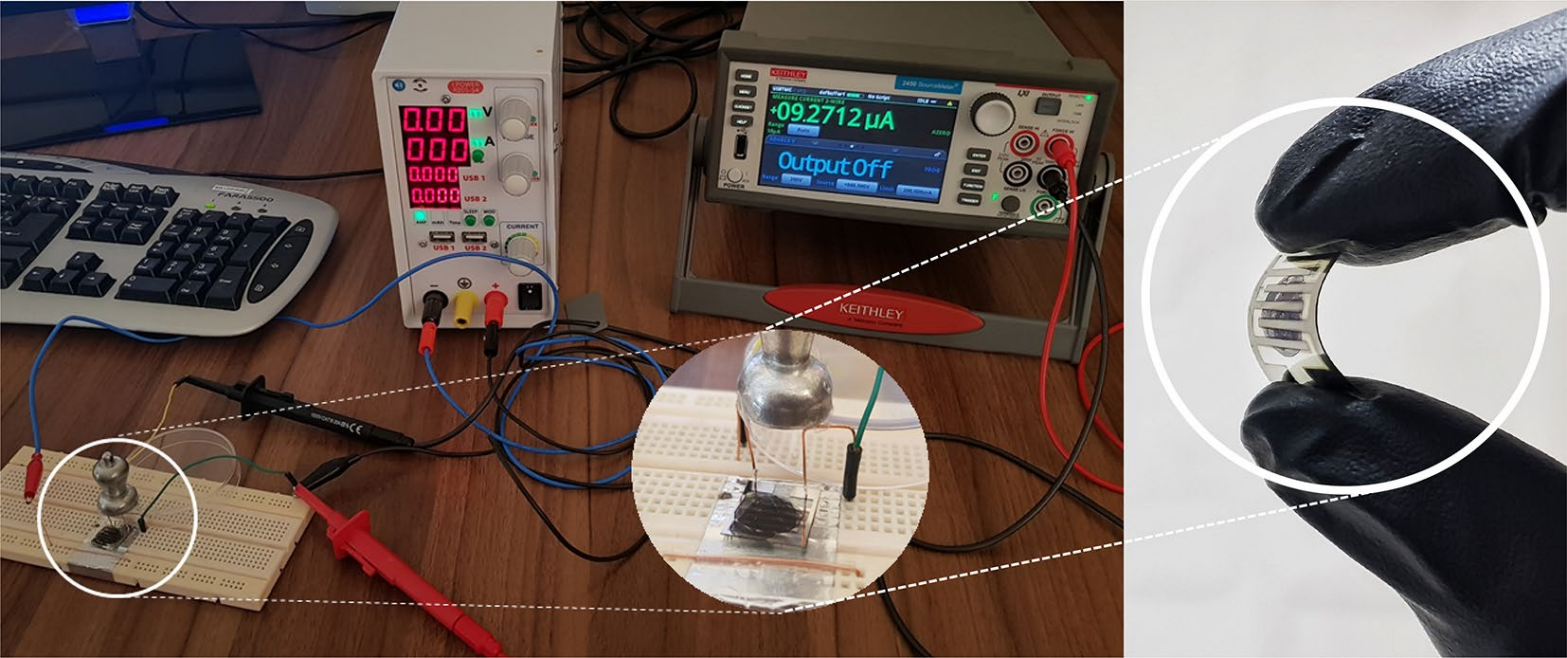
Photograph of electrical measurement set-up and the magnified photograph of the fabricated flexible biosensor.

In order to investigate the effect of the back gate voltage on the sensitivity of the fabricated biosensor, we compared the response to glucose in two sensing modes, one with the floating gate and the other with a biased gate. The flexible sensor and the measurement setup has been shown in Fig. 10.

**Figure 11:**
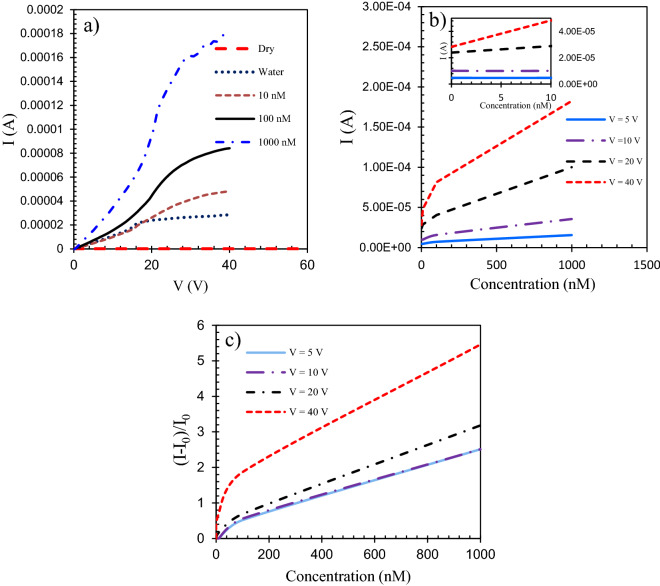
The electrical characteristic (I-V) of the resistive sensor in the presence of glucose from 10 nM to 1 µM. (**a**) I as a function of V. (**b**) I as a function of glucose concentrations for different voltages. (**c**) Sensor response (I_n_ − I_o_)/I_o_ as a function of glucose concentrations for different voltages.

First, we obtained the electrical characteristics of the device with the floating gate as a resistive glucose biosensor. The voltage is swept from 0 to 40 V and the current was recorded when the resistive biosensor was exposed to different glucose concentrations. Figure 11a shows the measurement results. According to the current–voltage (I–V) characteristic curves, as expected, the current of the biosensor increased with the increase of the voltage. Since the current difference for successive glucose concentrations increases with increasing the voltage, it can be concluded that the sensitivity increases in larger voltages. The electrical current-concentration (I-n) characteristic of the resistive biosensor has been plotted at V = 5, 10, 20, and 40 V in Fig. 11b. Based on the result, I_ds_ increase by adding glucose and the higher the glucose concentration the higher the current of the resistive sensor.

The plotted relative current change of the sensor in response to the glucose concentration changes is shown in Fig. 11c. The relative current change is defined as (I_n_ − I_o_)/I_o_ where I_o_ is the current of the sensor in the absence of the glucose and I_n_ is the sensor current in presence of the glucose concentration (*n*). The effect of the voltage on the relative current change of the BGFET sensor has also been investigated. As shown in Fig. 11c, the values of the relative current change, (I_n_ − I_o_)/I_o_, have been calculated and plotted in different drain voltages V = 5 V, 10 V, 20 V, and 40 V in the absence and the presence of glucose and for different glucose concentrations. Based on the results shown in Fig. 11c, the relative current change in V = 5 V, 10 V is equal and it is higher for higher drain voltages of V = 20 V, 40 V.

### Electrical characteristics of the fabricated flexible BGFET glucose sensor:transistor mode

**Figure 12:**
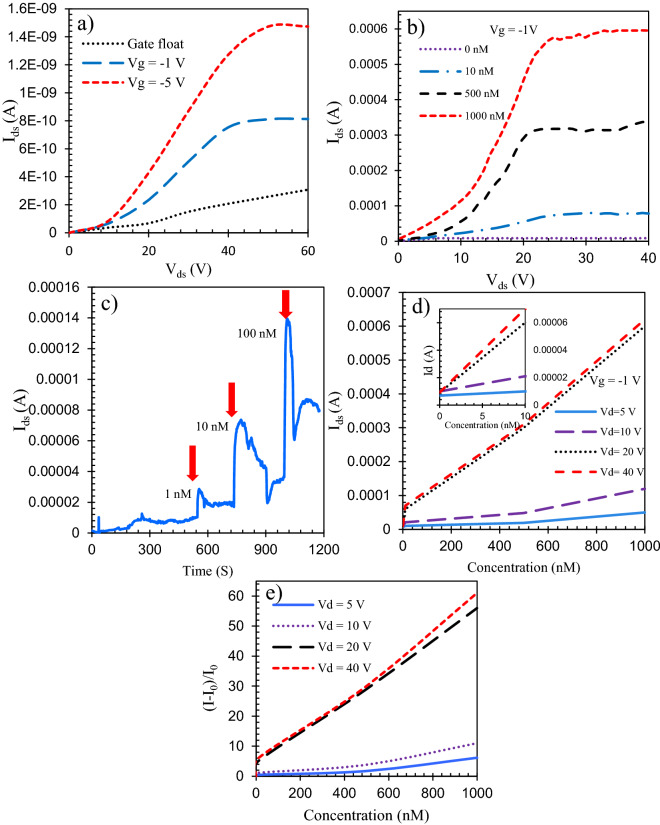
(**a**) I_ds_ as a function of V_ds_ without glucose for different gate voltages. (**b**) I_ds_ as a function of V_ds_ for different glucose concentrations including very low concentrations. (**c**) The very fast response of the sensor to the variations of glucose concentrations. (**d**) I_ds_ as a function of glucose concentrations for different drain voltages. (**e**) The relative current change as a function of glucose concentrations for different drain voltages.

The electrical characteristics of the proposed flexible BGFET with an rGO/CuO-NHS channel were tested primarily as a pure transistor in the absence of the glucose solution. The transfer characteristic (I_ds_ − V_ds_) is shown in Fig. 12. According to the output characteristic curves (Fig. 12a), the I_ds_ of the FET increases in response to the larger gate voltages (negative voltages) which indicates that the rGO/CuO-NHS has a p-type channel. In source-drain voltages between 0 and 40 V (for V_gs_ =  − 1 V), the I_ds_ increases linearly with the increase of V_ds_ that showing the linear (Ohmic) behavior of the transistor in a wide voltage range. At drain-source voltages greater than 40 V, the FET enters the saturation region in which the current does not change anymore by increasing V_ds_. The acquired electrical characteristics are plotted in Fig. 12b that show the rGO/CuO–NHS FET-based biosensor is capable of responing to the extraordinary (very low) glucose concentrations. So as to analyze the impact of the drain voltage on the overall performance of the FET in each transistor operation region (cut-off, linear, and saturation), V_ds_ was swept from 0 to 40 V and the drain current of the FET was recorded within the presence of glucose as depicted in Fig. 12b. As can be seen, the I_ds_ will increase with the augment of glucose concentration. The amperometric tests were started with a pure PBS solution as the analyte to be detected on the FET sensor operating in the saturation region. Then, the glucose solutions with different concentrations were dropped on the channel (Fig. 12c). The transistor current is increased by adding glucose and the sensor has a very fast response time. As the subsequent step, to acquire the suitable operating point of the transistor, the drain current for specific glucose concentrations has been measured for V_ds_ = 5 V, 10 V, 20 V, 40 V (V_gs_ =  − 1 V) which cover both the saturation and the linear regiona as shown in Fig. 12d. It can be concluded that for a fixed gate voltage, by increasing the V_ds_, both the I_ds_ and the sensitivity of the transistor (slope of the curves) increase. From Fig. 12d it can be deduced that the sensor has the most sensitivity inside the saturation region. Since the current values and the sensitivities are very near in V_ds_ = 20 V and 40 V, a drain voltage of 20 V (V_gs_ = − 1 V) has been selected as the operating bias of the FET. It is the lowest drain voltage that results in the highest sensitivity. The proposed sensor has also a very wide detection range with high sensitivity. We plotted the relative current change of the transistor in response to the glucose concentration changes in Fig. 12. The effect of the drain voltage on the relative current change ((I_n_ − I_o_)/I_o_) of the flexible FET has also been investigated. The values of the relative current change in different drain voltages V_ds_ = 5 V, 10 V, 20 V, 40 V (V_gs_ =  − 1 V) in the absence and the presence of glucose have been calculated and plotted for different glucose concentrations. As shown in Fig. 12e, I_ds_ and consequently the relative current change of the FET sensor are larger for higher drain voltages.

### The sensing mechanism of the sensor

The working principle of the glucose sensing of our proposed rGO/CuO–NHS-based BGFET can be explained based on the chemical reactions and the transistor action. When glucose comes in contact with CuO–NHS, first, a half-oxidation reaction of Cu(II) to Cu(III) takes place:

1 $${\text{CuO}} + {\text{OH}}^{ - } \to {\text{ CuO}}\left( {{\text{OH}}} \right) + {\text{ e}} - .$$

In the following, a nonenzymatic oxidation–reduction reaction between the formed Cu(III) oxyhydroxide and the adsorbed glucose molecules occur:

2 $${\text{2CuO}}\left( {{\text{OH}}} \right) + {\text{glucose}} \to {\text{2CuO}} + {\text{gluconolactone}} + {\text{H}}_{{2}} {\text{O}},$$

3 $${\text{Gluconolactone}} \to {\text{Glucononic acid}} \left( {{\text{Hydrolysis}}} \right).$$

**Figure 13:**
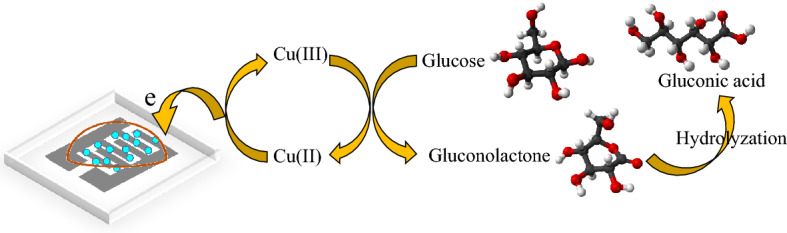
Schematic of the sensing mechanism of the rGO/CuO–NHS BGFET sensor.

It is observed that the oxidation of glucose and the reduction of metal oxyhydroxide CuO(OH) take place when CuO(OH) reacts with glucose^64,65^. During this reaction, the glucose is oxidized into gluconolactone, the CuO(OH) is reduced into CuO, and the free-electron is continuously produced. Since CuO–NHs have a larger surface to volume ratio than the bulk CuO, a large number of electrons will be transferred to the drain-source electrodes from the solution. In this process, graphene nanosheets enhance the electrocatalytic activity of glucose oxidation which leads to the production of a larger number of electrons and the increase in the conductivity of the channel that result in a higher FET current. The gate of the FET amplifies the produced current so that a higher current can be read for small concentrations of glucose compared to a resistive device. The number of the electrons are also increased with the increase in the concentration of glucose because of the rise of the previously mentioned phenomena. The schematic of the sensing mechanism of the rGO/CuO–NHS BGFET sensor is shown in Fig. 13.

### Effects of environmental parameters

**Figure 14:**
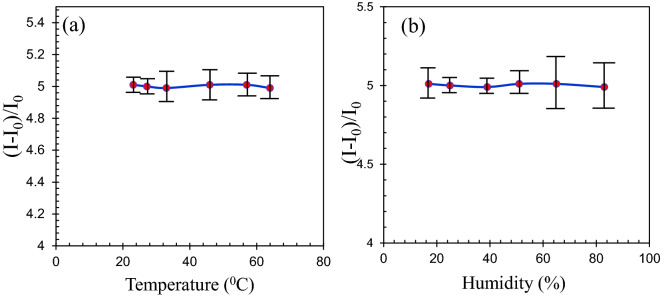
Effect of (**a**) temperature and (**b**) humidity on the sensor performance in response to 5 nM glucose.

The effects of environmental parameters such as temperature and humidity on FET performance are studied as well. The enzymatic glucose sensors suffer from poor stability resulted from variations of operating temperatures, pH values and relative humidity^53,66^. We have shown that the proposed sensor is stable and its performance does not depend on environmental conditions. The effect of temperature is investigated by varying the temperature from 23 to 62 °C in response to 5 nM glucose in Fig. 14a. As can be seen, the relative current change response of sensor is nearly constant with increasing the temperature.

The proposed sensor benefits from the advantages of non-enzymatic glucose sensors and because of its FET based mechanism, it is free from the problems associated with the electrochemical sensors. Electrochemistry biosensors, because of their the enzymatic activity, are sensitive to changes in temperature^66,67^. That is, by increasing the temperature, the kinetic energy of GOx increases and thus more glucose are oxidized producing an increased number of electrons.

The effect of relative humidity (RH) is also studied by varying the humidity from 17 to 83% as shown in Fig. 14b. Figure 14b showed that the sensor can maintain its performance in high humidity conditions. The sensors were stored 2 h in hot and humid condition and they were able to maintain their performance with a high stability.

### Sensitivities

In this part, sensitivities (S) for the corresponding outputs in resistive and transistor sensing modes have been calculated and compared. The obtained outputs were plotted in Figs. 11b and 12d. The sensitivities are calculated as the slope of each I–n graph for the almost linear detection ranges. The sensitivity for current output is defined as:

4 $$S=\frac{\Delta I}{\Delta n}=\frac{ {\mathrm{I}}_{n}-{\mathrm{I}}_{0}}{\Delta n}.$$

The sensitivity of the resistive biosensor towards glucose from 0 to 1 µM with a detection limit of 10 nM has been calculated as 151.7 μA μM^−1^. The sensitivity of the FET towards glucose from 0 to 1 µM with a very low detection limit of 1 nM has been calculated as 600 μA μM^−1^.

Based on the obtained results, the use of the field-effect transistor as the bioelectronic sensor has advantages such as higher sensitivity and lower limit of detection compared to the resistive sensor.

### Repeatability, stability, reproducibility and selectivity

**Figure 15:**
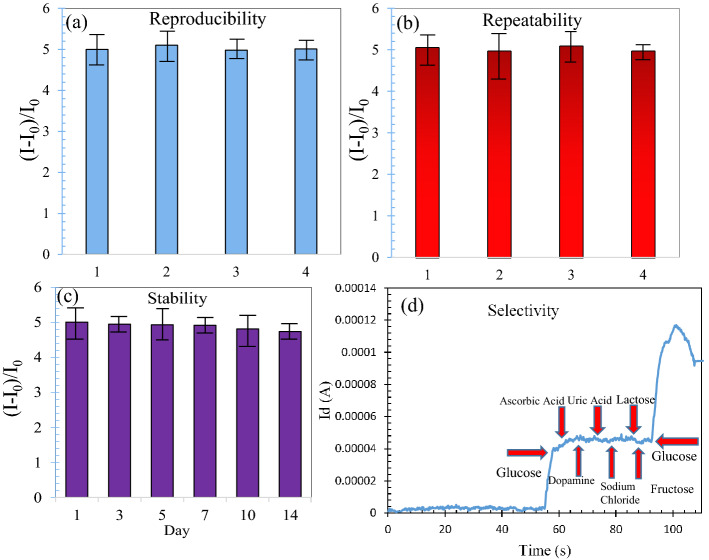
(**a**) Repeatability, (**b**) reproducibility, (**c**) stability, and (**d**) selectivity of the flexible FET sensor.

The repeatability was examined via four similar measurements by the same sensor as shown in Fig. 15a. Almost all the responses are the same to 5 nM glucose. The RSD of the results in Fig. 15a is obtained 0.6%, indicating the extremely good repeatability of the proposed biosensor. We investigated the repeatability measurements with three other fabricated sensors. All of them showed a very good repeatability with the RSDs of 0.5%, 0.65%, 0.7%.

The reproducibility of the fabricated sensor was investigated by fabricating three BGFETs with the same fabrication procedure and measuring their output currents in reaction to the 5 nM glucose. The responses of FETs have been measured and shown in Fig. 15b. The comparison of the three sensors shows that they exhibit similar behavior and they are almost the same. The stability of the biosensor is examined by using one sensor three times a week for 2 weeks. The sensor responses confirmed high stability in cycles of measurements and retained 93% of the preliminary response value after 2 weeks (Fig. 15c). To analyze the selectivity of the proposed BGFET glucose sensor, its overall performance is examined in the presence of 5 nM glucose and 1 nM of each possible interfering species (ascorbic acid, sodium chloride, uric acid, lactose, fructose, and dopamine). Figure 15d shows that the sensor response to glucose is selective and it does not react to the added interfering species.

### Human blood sample tests

The excellent, reproducible and stable results of the FET towards the glucose, motivated us to quantitatively test the real human blood serum samples. We used five samples of serums with different blood sugar levels. The serum samples were analyzed using DIRUI CS-800 Auto Chemistry Analyzer in a pathobiology laboratory and the measured values (4.29, 5.29, 6.02, 7.80, 12.65 mM glucose) were compared with the obtained values by our fabricated FET as illustrated in Table 1. The measurements showed that the glucose values measured by the proposed sensor in our lab are in very good agreement with the values obtained from the commercial instrument. Thus, as the measurement results verify, the FET based on rGO/CuO-NHS can be used as a potential device for glucose detection in real samples.

**Table 1:** Comparison of glucose values obtained by the commercial instrument with the values measured by the FET-based biosensor.

Values measured by commercial instrument (mM)	Values measured by FET (mM)	RSD (%)	Recovery (%)
4.29	4.2	1.06	97.90
5.29	5.37	0.75	101.51
6.02	6.12	0.82	101.66
7.80	7.91	0.7	101.41
12.65	12.53	0.47	99.05

In Table 2 we have compared the recently reported glucose sensors with the results obtained from our rGO/CuO-NHS flexible FET sensor. As can be seen, the proposed glucose biosensor showed a high sensitivity (600 μA μM^−1^) and the lowest detection limit of 1 nM which indicates its impressive capability in the detection of extremely low glucose concentrations (0–1 µM). The results discussed in this work showed that the proposed biosensor using can be a promising alternative to the electrochemical test strips. The stabilized fabrication process and the flexibility of the proposed sensor make it a suitable choice for wearable applications. It can also be used as a glucose detection device employing body fluids such as saliva which is considered for our future work. Because of its excellent, sensitive, selective, and stable current response, the flexible FET with the hybrid material of rGO/CuO–NHS as the channel has a promising potential to be used as portable blood glucose sensors.

**Table 2:** Comparison of the proposed glucose sensor with other reported sensors.

Sensing material	Sensitivity	LDR	DL	T-FET	Year	Ref
LICNT	0.302 mV mg^−1^ dl^−1^	60–360 mg^−1^ dl^−1^	60 mg^−1^ dl^−1^	EGFET	2013	^68^
Graphene	2.5 mA mM^−1^	0.1–10 mM	0.1 mM	SGFET	2014	^4^
ZnO NRs	32.27 μA mM^−1^ cm^−2^	0.05–70 mM	0.07 μM	SGFET	2015	^69^
MWCNTCOOH/PANI	nr	0.005–50 mM	0.005 mM	EGFET	2016	^70^
Nafion/GOx/ZnO NRs	22.3 µA mM^−1^ cm^−2^	0–80 mM	0.07 mM	EGFET	2017	^71^
Fe_2_O_3_-ZnO NRs	105.75 µA mM^−1^ cm^−2^	0.05–18 mM	0.012 mM	EGFET	2017	^72^
MoS_2_	260.75 mA mM^−1^	300 nM-30 mM	300 nM	BGFET	2018	^5^
ZnO NRs	1.6 mA μM^−1^ cm^−2^	nr	1 μM	BGFET	2018	^6^
In_2_O_3_	nr	10 nM-1 mM	10 nM	SGFET	2018	^21^
RuO_x_	6.89 mV mM^−1^	1 -8 mM	1 mM	ExGFET	2019	^10^
Graphene/Ag	9.9596 μA μM^−1^	0.1–0.35 μM	0.0262 μM	SGFET	2020	^73^
CuO NRs	3.03 mV mM^−1^	1–12 mM	1 mM	EGFET	2020	^7^
Graphene	nr	0.05–100 mM	0.15 μM	SGFET	2020	^74^
Ni/Cu-MOFs	26.05 μA mM^−1^ cm^−2^	1 μM–20 mM	0.51 μM	EGFET	2021	^75^
CuO/ZnO NRs	6.643 mV mM^−1^	1–8 mM	1 mM	ExGFET	2022	^76^
rGO/CuO-NHS	600 μA μM^−1^	0–1 µM	1 nM	BGFET	2023	This work

*LDR* linear dynamic range, *DL* detection limit, *T-FET* type of FET, *Ref* References, *LICNT* Laser-irradiated carbon nanotube thin films, *ZnO NRs* ZnO nanorods, *MWCNTCOOH/PANI* multi-walled carbon nanotubes-polyaniline nanocomposite, *Fe*_*2*_*O*_*3*_*-ZnO NRs* Iron(III) oxide-ZnO nanorods, *MoS2* molybdenum disulfide, *Pd/Graphene* Palladium-graphene nanocomposite, *RuO*_*x*_ ruthenium oxide, *Graphene/Ag* silver-graphene nanocomposite, *CuO NRs* CuO nanorods, *Ni/Cu-MOFs* bimetallic nickel-copper metal–organic frameworks, *nr* not reported.

Another comparative table, comparing the developed sensor to other commercially available electrochemical sensors by comparing LOD, response time and cost is shown in Table 3. Table 3 shows our FET based test strip has a lower LOD, faster response time and an acceptable cost in comparison with other commercially available test strips.

**Table 3:** Comparing the developed sensor to other commercially available electrochemical strips.

Glucometer brand	LOD	Response time (s)	Cost (per strip)
On.Call® Vivid	0.6 mM	5	$0.5
ReliOn	1.1 mM	7	$0.29
OneTouch Ultra	1.1 mM	5	$1
ACCU-CHEK	0.6 mM	4	$0.45
Bayr contour next	1.1 mM	5	$0.38
FET based on rGO/CuO	1 nM	3	$1

## Conclusions

A flexible sensor based on rGO/CuO–NHS was fabricated. Ag electrodes on both sides of a PET substrate have been used to design a back gate FET and a resistive biosensor. The hybrid material was used as the channel of the FET that served as the sensitive material to glucose. The electrical characteristics of the transistor and the resistive biosensors were measured before and after the presence of glucose. The current of the biosensor increased with the addition of the glucose concentrations. The BGFET sensor could measure the glucose concentrations in a range from 0 to 1 µM with a very low detection limit of 1 nM. The fabricated rGO/CuO–NHS FET showed an excellent sensing performance, high selectivity, sensitivity, and stability. The sensitivity of the FET towards glucose was calculated about 600 μA μM^−1^ in the range from 0 to 1 µM. The responses of the FET-based biosensor to the human blood serum samples were verified in a Pathology laboratory and were in very good agreement with the commercial device. Due to the high accuracy and the high selectivity of the proposed sensor, it has the capability of being used in point-of-care applications in near future.

## Methods

### Blood serum samples

Blood serum samples were from Shahid Beheshti Hospital. All experiments and methods were performed in accordance with relevant guidelines and regulations. All experimental protocols were approved by the ethics committee of the laboratory of Shahid Beheshti Hospital. Informed consent was obtained from all subjects.

### Materials

D(+) glucose, graphene oxide, and Cu(CH_3_COO)_2_·H_2_O were purchased from Sigma Aldrich.

### Apparatus

The structural and morphological analysis of rGO/CuO–NHS was investigated using X-ray diffraction (BRUKER D8 Advanced instrument), scanning electron microscopy (TESCAN Mira3 device) and Atomic Force Microscopy (ARA-AFM device). The pore size and distribution were analyzed by DLS method performed by Horiba SZ-100. The Fourier transform infrared (FTIR) spectra of rGO/CuO–NHS was recorded using BRUKER ALPHA FTIR device. The surface area, the pore size, and the pore size distribution of the rGO/CuO-NHS were obtained from the nitrogen adsorption–desorption isotherms using the Brunauer–Emmett–Teller (BET) and Barrett–Joyner–Halenda (BJH) methods using BELSORP-mini X instrument. The silver drain and source electrodes were transferred on PET substrate by DC sputtering. The drain-source current of the flexible transistor and resistive biosensor were measured by Keithley source measure unit and the back gate voltages were applied by laboratory dc power supply (GPS-3306D).

### Synthesis of materials

CuO hollow-spheres decorated with the reduced graphene oxide are synthesized by the hydrothermal method. Firstly, 0.66 g Cu(CH3COO)_2_·H_2_O and 25 mg graphene oxide (GO) were dissolved in 40 ml of deionized water under vigorous stirring for 1 h. Afterward, the mixture was transferred into a 50 ml stainless steel autoclave, which was sealed and kept at 160 °C for 12 h in an oven. After cooling to room temperature, the black product was percolated and washed several times with deionized water and ethanol, and finally dried in the oven overnight.

### Fabrication of flexible FET sensor

The process of fabrication and the biasing voltages of the BGFET sensor is schematically plotted in Fig. 3a. The measurement set-up and the flexible BGFET have been shown in Fig. 4. Polyethylene tetraphtalate (PET) substrate has features like flexibility, thermal resistance and mechanical strength. The flexible sensors were fabricated on a PET substrate with a thickness of 0.175 mm. The PET substrates was cleaned with DI water, ethanol and acetone. First, 250 nm thick silver electrodes were deposited on the surface of a flexible PET substrate (dimensions of 1.5 × 2 × 0.175 cm^3^) by using DC sputtering method via a patterned paper shadow mask. The deposited electrodes were designed in the form of interdigitated contacts with a 1 × 1 cm^2^ sensing area containing 6 fingers, 900 µm gap spacing, and 1000 µm finger widths (Fig. 3b). The shadow mask was in contact with the substrate to prevent the blurring which is a common issue in shadow mask lithography. Blurring can cause broadening of the geometrical dimensions of the evaporated silver electrodes on the substrate with respect to the shadow mask feature. On the other facet of the PET substrate, a thin layer of indium tin oxide (ITO) was deposited so that it can manipulate the electric charge carriers’ transport inside the channel between source and drain electrodes. To prepare the sensitive channel of the transistor, 100 mg of CuO/rGO composite was dissolved in 10 ml of ethanol and sonicated for 5 min to obtain a homogenous solution. Then, the sensor has been on the hotplate at 60 °C and 80 µl of the CuO/rGO solution is deposited by drop-casting technique. This process has been repeated two times. Each time 40 µl of CuO/rGO solution was dropped on the surface of the silver source and drain electrodes via micropipette and it was allowed for 2 min to dry. The concentration of the deposited CuO/rGO channel was 10 mg/ml. This way, a flexible BGFET-based sensor was fabricated with the silver interdigitated drain and source electrodes, the ITO back gate, and a channel of rGO/CuO–NHS for glucose detection.

## Data Availability

The datasets generated and analysed during the current study are available from the corresponding author on reasonable request.
